# Reducing unnecessary caesarean sections: scoping review of financial and regulatory interventions

**DOI:** 10.1186/s12978-020-00983-y

**Published:** 2020-08-31

**Authors:** Newton Opiyo, Claire Young, Jennifer Harris Requejo, Joanna Erdman, Sarah Bales, Ana Pilar Betrán

**Affiliations:** 1 UNDP/UNFPA/UNICEF/WHO/World Bank Special Programme of Research, Development and Research Training in Human Reproduction (HRP), Department of Reproductive Health and Research, World Health Organization, Avenue Appia 20, 1211, Geneva 27, Switzerland; 2 Data and Analytics Section, Division of Data, Analytics, Policy and Monitoring, UNICEF USA, New York, USA; 3 Department of Epidemiology of Microbial Disease, Yale School of Public Health, New Haven, CT USA; 4 Schulich School of Law, Dalhousie University, Halifax, Canada; 5 Hanoi University of Public Health, Hanoi, Vietnam

**Keywords:** Scoping review, Caesarean section, Overuse, Payment mechanisms, Financial interventions, Regulatory interventions, Legislative interventions

## Abstract

**Background:**

Caesarean sections (CS) are increasing worldwide. Financial incentives and related regulatory and legislative factors are important determinants of CS rates. This scoping review examines the evidence base of financial, regulatory and legislative interventions intended to reduce CS rates.

**Methods:**

We searched MEDLINE, EMBASE, CINAHL and two trials registers in June 2019. Both experimental and observational intervention studies were eligible for inclusion. Primary outcome measures were: CS, spontaneous vaginal and instrumental birth rates. We assessed quality of evidence using Grading of Recommendations, Assessment, Development and Evaluation (GRADE) method.

**Results:**

We identified 9057 articles and assessed 65 full-texts. We included 16 observational studies. Most of the studies were conducted in high-income countries.

Three studies assessed payment methods for health workers: equalising physician fees for vaginal and caesarean delivery reduced CS rates in one study; however, little or no difference in CS rates was found in the remaining two studies.

Nine studies assessed payment methods for health organisations:

There was no difference in CS rates between diagnosis-related group (DRG) payment system compared to fee-for-service system in one study. However, DRG system was associated with lower odds for CS in another study.

There was little or no difference in CS rates following implementation of global budget payment (GBP) system in two studies.

Vaginal birth after caesarean section (VBAC) increased after implementation of a case-based payment system in one study. Caesarean section increased while VBAC rates decreased following implementation of a cap-based payment system in another study.

Financial incentive for providers to promote vaginal delivery combined with free vaginal delivery policy was found to reduce CS rates in one study.

Studied regulatory and legislative interventions (comprising legislatively imposed practice guidelines for physicians in one study and multi-faceted strategy which included policies to control CS on maternal request in another study) were found to reduce CS rates.

The GRADE quality of evidence varied from very low to low.

**Conclusions:**

Available evidence on the effects of financial and regulatory strategies intended to reduce unnecessary CS is inconclusive given inconsistency in effects and low quality of the available evidence. More rigorous studies are needed.

## Plain English summary

Caesarean sections are increasing worldwide. Financial incentives and related regulatory and legislative factors are important determinants of caesarean section rates. Financial interventions may influence caesarean section rates in various ways: for example, equalizing the payment between caesarean and vaginal delivery may reduce health provider incentive to perform unnecessary caesarean section; increasing the cost of caesarean section to patients may disincentivize women from choosing medically unnecessary caesarean section.

However, in previous systematic reviews of non-clinical interventions intended to reduce unnecessary caesarean section, several financial, regulatory and legislative interventions were excluded due to weaknesses in study designs. The real-world effect of these interventions therefore remain unclear. This scoping review addresses this gap in the literature.

We searched multiple databases for published, unpublished and ongoing studies in June 2019. Sixteen studies met our predetermined inclusion criteria and were included in the review.

Most of the studies assessed financial interventions comprising: different payment methods for health workers (such as equalizing physician fees for vaginal and caesarean deliveries); different payment methods for health organisations (such as budget systems where the hospital is given a fixed amount of money in advance to cover expenses for a fixed period). The quality of evidence varied from very low to low. Overall, studied interventions resulted in mixed-effects on caesarean section rates.

In summary, the available evidence on the effects of financial and regulatory strategies intended to reduce unnecessary caesarean section rates is inconclusive given inconsistency in effects and low quality of the available evidence. More rigorous studies are needed.

## Background

Caesarean section (CS) is a surgical procedure that saves lives when used for medically indicated reasons (e.g. abnormal presentation or fetal distress). Caesarean sections are increasing worldwide with wide differences in use within and between countries [1–3]. Latest trends analysis shows that between 2000 and 2015, the global average CS rate increased by 9.0% (from 12.1 to 21.1%) [4]. Latin America and the Caribbean had the highest CS rates (44.3%), followed by North America (32.0%), Middle East and North Africa (29.6%), East Asia and Pacific (28.8%), Eastern Europe and Central Asia (27.3%), Western Europe (26.9%), South Asia (18.1%), Eastern and Southern Africa (6.2%) and West and Central Africa (4.1%) [4]. The steady increase in CS has, however, not been accompanied by clear health or other benefits for women and their babies [5]. This evidence suggests that the majority of CS are medically unnecessary and that many women and their babies are without the benefits of vaginal birth [6, 7]. These advantages compared to CS include: women’s shorter physical and psychological recovery period after birth, increased likelihood of successful breastfeeding, natural physiological adaptation to the external environment and improved immunity of the baby, and support for the baby’s longer-term growth, health and development [8, 9].

Underuse and lack of access to CS, particularly among rural and disadvantaged communities in low resource settings, can result in unnecessary birth complications and deaths of women and their babies [10]. Conversely, overuse (use of CS without medically indicated reasons) is associated with increased risk of harm for women and their babies (e.g. infections, bleeding, complications related to use of anaesthesia, infant respiratory problems such as asthma, obesity in children and added complications for the women in subsequent pregnancies such as uterine rupture, abnormal placenta, miscarriage, stillbirth, post-traumatic stress disorder (PTSD), among others) [11–13]. Caesarean sections are expensive, and their overuse has resource implications particularly for countries where essential interventions remain unavailable, or where inequalities in access to these services remain. The steady increase in CS in the last decades coupled with the limited success of tested interventions to stop and reverse this trend have raised concerns among governments and health care professionals [6, 7].

A range of clinical and non-clinical interventions intended to reduce CS have been assessed in previous systematic reviews [14–16]. Financial factors and related regulatory and legislative factors are important determinants of CS practices [17, 18]. There are various pathways through which targeted financial interventions may reduce unnecessary CS and ultimately improve maternal and neonatal health outcomes: (1) reducing health provider incentives to perform unnecessary CS, by equalizing the payment between CS and vaginal delivery or increasing vaginal delivery fee higher than CS fee; (2) disincentivizing women from choosing medically unnecessary CS, by increasing the cost of CS to patients, or reducing the costs of vaginal deliveries (e.g. free vaginal delivery policies); (3) pay-for-performance, that involves rewarding hospitals for performance in reducing CS or increasing vaginal deliveries; (iv) global or standardised budget caps (including capitation budget) for all patients and all services, by reducing costs for entire patient populations, rather than for individual cases, including switching from CS to vaginal delivery to save on costs and ensure a higher overall surplus. Similarly, regulatory and legislative interventions may reduce unnecessary CS by reducing the discretion of individual clinicians to perform CS that are convenient for them, or that involve requests from patients in the absence of any maternal or fetal indications.

However, in previous systematic reviews of non-clinical interventions, several financial, regulatory and legislative interventions were excluded [15] due to weaknesses in study designs (e.g. cross-sectional, case study designs). The real-world impact of these interventions, often studied in ways not amenable to systematic reviews of interventions (e.g. case studies) therefore remain uncertain. Furthermore, factors that may influence the implementation of the interventions, including challenges and opportunities, remain understudied.

This scoping review examines the evidence base of financial, regulatory and legislative interventions intended to reduce unnecessary CS. The specific objectives are to:

- Describe the characteristics of identified financial, regulatory and legislative interventions intended to reduce unnecessary CS
- Assess the safety and effectiveness of these financial, regulatory and legislative interventions
- Summarise evidence on the lessons learnt on the design and implementation of identified financial, regulatory and legislative interventions to inform policy, implementation and future research.

## Methods

### Study selection criteria

#### Types of studies

Studies that assessed the effect of financial, regulatory and legislative interventions for reducing CS using the following designs were eligible for inclusion: randomised trials, non-randomised trial, controlled before-after (CBA) studies, interrupted time-series (ITS) studies, cohort studies, large scale intervention case studies, pre-post studies intervention studies, cross-sectional studies (with appropriate analysis to control for confounding), and mixed-methods (quantitative and qualitative) studies.

#### Types of participants

Studies involving the following groups were eligible for inclusion: (1) low-risk pregnant women seeking antenatal, labour and delivery care in health care facilities (term, singleton, cephalic pregnancies with or without a previous caesarean; women in Robson classification groups 1 through 5 [19]); (2) healthcare providers who work with pregnant women (nurses, midwives, physicians) during antenatal care, labour or delivery; (3) healthcare facilities, hospitals or health systems that provide delivery care to pregnant women.

#### Types of interventions

Financial, regulatory and legislative interventions eligible for inclusion are summarised in Table 1. Studies of interventions aimed at increasing CS use were excluded.

**Table 1:** Financial, regulatory and legislative interventions eligible for inclusion

Strategy	Details of strategy
**a) Financial interventions**	
***Payment methods for health workers***	
Capitation	Prospective*, fixed payment to healthcare providers in order to care for a defined population for a defined period of time such as a year. Payment made to physicians per patient registered with them or in their care. Reimbursement for providers is not linked to inputs (such as diagnostic tests) or to the volume of service provided to patients. Physician knows in advance the amount of payment they will receive before any care is provided. This prospective payment plan may encourage healthcare providers to contain costs. *The term prospective refers to when the payment rate for a predefined package of healthcare services for a fixed period of time is determined before the treatment takes place.
Equalizing reimbursement fees for vaginal and caesarean deliveries	Equalising fees intended to reduce incentives / motivations for healthcare providers to perform CS.
Higher reimbursement fees for vaginal than caesarean deliveries	Higher fees for vaginal deliveries intended to motivate health care providers to switch from caesarean to vaginal deliveries in the absence of medical justification.
***Payment methods for health organisations***	
Global budget payment (GBP) systems	Aggregate cash sum, fixed in advance by payers/government agency, intended to cover (reimburse) the total cost of a service provided, and is usually set for 1 year ahead. The hospital, health facility or clinic is used as a unit of payment based on previous historical spending, the volume of service and hospital bed size, which are brought together in a resource allocation formula. GBP system is intended to create incentives for hospitals to reduce the volume of services provided (including reducing unnecessary services) and encourage efficient resource utilisation.
Case-based reimbursement systems	A prospective reimbursement mechanism in which hospitals are paid for each discharged inpatient case, based on a previously defined rate for each group of cases with similar clinical conditions and resource requirement. The International Classification of Diseases (ICD) developed by WHO is widely used to define these groups for the purpose of setting payment rates. Hospitals are paid a fixed payment rate for each treated case that falls into one of a set of defined categories of cases, such as diagnosis-related groups (DRGs). Case-based payment mechanism provides significant incentives for providers to contain cost per case by minimising the use of resources utilised per case, for example, reducing the unnecessary utilisation of diagnostic and surgical services.
Diagnosis related group (DRG) reimbursement schemes	Diagnosis-related-groups (DRGs) are clinically meaningful groups of patients with similar clinical characteristics and resource consumption patterns who incur comparable costs. DRGs provide a flat per-discharge payment that varies based on diagnosis, severity, and the procedures performed. All patients treated by a hospital are classified into a limited number of DRGs, which are supposed to be clinically meaningful and relatively homogenous in their resource consumption patterns. Each DRG is associated with a specific cost weight or tariff, which is usually calculated from information about average treatment costs of patients falling within a specific DRG in at least a sample of other hospitals in the past. Depending on the country, hospitals under DRG-based hospital payment systems either receive a DRG-based case payment or a DRG-based budget allocation.
Co-payment	Direct patient payments for part of the cost of health services. Copayment when CS is not medically indicated is intended to disincentivize women from choosing medically unnecessary CS, by increasing the cost of CS to patients).
**b) Regulatory and legislative interventions** Includes interventions by the state to avoid unnecessary caesarean sections. Intervention can take various forms including taxes, strategic policy guidance, performance contracts, or legal rules that seek to improve compliance of health care professionals or hospitals with national policies on evidence-based obstetric practice.	
Liability of health care organisations	Interventions that limit liability of health care organisations, for example malpractice lawsuits (legal cover may reduce CS conducted for fear of medical lawsuits).
Insurance	Regulation of the provision of insurance, for example insurance coverage of maternal health services (may influence women’s requests for non-medically indicated CS, particularly if they do not bear the full cost through insurance).
Quality of practice	Interventions for assuring quality of care, for example legislatively imposed clinical guidelines may reduce unnecessary CS by reducing the discretion of individual clinicians to perform CS that are convenient for them, or that involve requests from patients in the absence of any maternal or fetal indications.
Professional competence	Interventions for assuring professional competence (for example, accreditation requirements intended to improve compliance with evidence-based obstetric practice may reduce non-medically indicated CS).
Professional liability	Regulation of liability for health professionals (for example, provision of legal cover may reduce CS conducted for fear of medical lawsuits).
**c) Others (as reported in identified eligible studies)**	
Source: Effective Practice and Organisation of Care (EPOC). EPOC Taxonomy; 2015. epoc.cochrane.org/epoc-taxonomy (Accessed 26 Dec 2019)	

#### Types of outcome measures

##### Main outcomes

Rate of CS and rate of all other modes of delivery (spontaneous vaginal birth, elective CS, emergency CS, instrumental vaginal birth).

##### Secondary outcomes

Perineal/vaginal trauma, birth trauma, perinatal asphyxia, serious maternal morbidity, minor maternal morbidity, long-term maternal outcomes, long-term infant outcomes, maternal birth experience, health care resource utilization (Table 2).

**Table 2:** Primary and secondary outcome measures

Primary outcomes	
**Rate of caesarean section and rate of all other modes of delivery** (spontaneous vaginal birth, caesarean section before labour, emergency caesarean section, instrumental vaginal birth)	
**Secondary outcomes**	
**•Perineal/vaginal trauma** (including 2nd, 3rd, or 4th degree perineal tears, obstetric anal sphincter injuries (OASIS), vaginal tears, episiotomy, perineal suturing, postpartum perineal pain) **•Birth trauma** (fractured skull, haematoma, cerebral haemorrhage, fractured clavicle, facial paralysis, brachial plexus injury, scalp injury, facial skin lesions, retinal haemorrhage) **•Perinatal asphyxia** (low Apgar score at 5 min, cord blood acidosis, needed major resuscitation (respiratory support, intubation at birth), hypoxic ischaemic encephalopathy) **•Maternal birth experience** (including maternal satisfaction with care, women’s mental and psychological health assessment, rating of birth experience, or as defined by study authors)	**•Maternal morbidity** (including febrile morbidity, peripartum infection, wound complication, post-partum hemorrhage, or as defined by authors) **•Long-term infant outcomes** (breastfeeding, childhood disability, mother-infant bonding/separation) **•Serious maternal morbidity** (including organ failure, obstetric hysterectomy, sepsis, severe obstetric haemorrhage (antepartum or postpartum), uterine rupture, admission to intensive care or as defined by trial authors) **•Long-term maternal outcomes** (including urinary or faecal incontinence, obstetric fistula, utero-vaginal prolapse) **•Health care resource utilization** (length of hospital stay, maternal readmission/ rehospitalisation, readmission/ rehospitalisation in neonatal period (up to 28 days), cost of care, referral for higher level care)

Studies that only reported secondary outcomes were not included.

### Settings

All settings (Low, Middle, High-income countries).

### Search methods for identification of studies

The following sources were searched in June 2019 for eligible published, unpublished and ongoing studies (Additional file 1): MEDLINE, EMBASE, CINAHL, GIM (Global Index Medicus; http://www.globalhealthlibrary.net), Ebsco MultiDisciplinary Databases (http://www.ebsco.com), WHO International Clinical Trials Registry Platform (http://www.who.int/ictrp/en/), ClinicalTrials.gov (https://www.clinicaltrials.gov), and reference lists of identified trials and reviews. The searches were done without any language, date or publication status restrictions.

### Data collection and analysis

#### Selection of studies and data extraction

The identified articles were entered in Covidence (https://www.covidence.org/). Three reviewers (NO, CY, APB) independently screened titles, abstracts and full texts of identified articles and applied the pre-specified study eligibility criteria to select studies. Two reviewers (NO, CY) extracted and entered data (e.g. on study settings, participants, interventions, outcome measures) independently and in duplicate into pilot-tested data extraction forms. Disagreements were resolved by discussion.

#### Assessment of quality of included studies

Risk of bias in each study was assessed independently and in duplicate by two reviewers (NO, CY) and reported as one element of Grading of Recommendations Assessment, Development and Evaluation (GRADE) [20]. Risk of bias domains assessed included the likelihood of bias attributable to confounding, selection of participants in the study, classification of interventions, deviations from intended interventions, missing data, measurement of outcomes, and selection of reported results.

The certainty of the body of evidence for each outcome (sometimes referred to as *“quality of evidence”* or *“confidence in the estimate”*) was assessed using the GRADE approach [20]. Based on this approach, the certainty of evidence for each outcome is rated as “High”, “Moderate”, “Low” or “Very low” based on a set of criteria. The certainty of evidence from randomised controlled trials (RCTs) begin at “High” and can be downgraded in consideration of five factors: risk of bias or limitations in the design and conduct of study, directness of evidence, consistency of results, precision of effect estimates and publication bias. Certainty of evidence from observational studies begin at “Low” and can be upgraded in consideration of three factors: magnitude of effect estimate, dose-response gradient, and influence of residual plausible confounding.

### Data synthesis

We did not combine results using statistical methods (meta-analysis) as the included studies utilised varied designs (RCTs, controlled before-after, interrupted time series, pre-post intervention studies) and examined diverse interventions (financial, regulatory, legislative strategies). Individual study results are described in the Results section.

For interrupted time series studies, we reported two effect sizes used to measure the intervention effect: *change in level* (also called “step change”) and *change in trend* (also called “change in slope”) before and after the intervention [21]. Change in level is the difference between the observed level at the first intervention time point and that predicted by the pre-intervention time trend; change in trend is the difference between post- and pre-intervention slopes. A negative change in level and slope indicates a reduction in the event. Where these effect measures were not estimable (e.g. owing to insufficient data), we reported results as reported in the individual studies.

Data on lessons learnt (relating to features, challenges, and opportunities in the design and implementation of studied interventions) were collated based on reports from the included studies.

## Results

### Results of the search

**Fig. 1:**
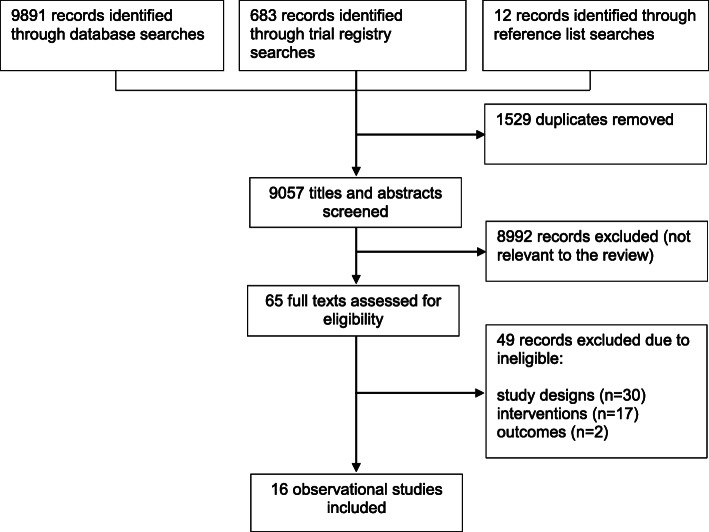
Results of the literature search and studies selected

We identified 9057 records from electronic databases, clinical trials registries and other resources. We excluded 8992 records following a review of titles and abstracts. We retrieved the full texts of the remaining 65 records for detailed eligibility assessment. We excluded 49 records due to ineligible interventions (e.g. user fee exemptions designed to increase CS), non-intervention studies and inappropriate outcomes. Overall, 16 studies fulfilled the review inclusion criteria (Fig. 1).

### Included studies

The sixteen included studies form the basis of the findings summarised in this review (Table 3). These studies were conducted in six different countries: North America (5 studies in USA); Latin America (1 study in Brazil); Western Asia (1 study in Iran); East Asia (2 studies in China; 5 studies in Taiwan; 2 studies in South Korea).

**Table 3:** Characteristics of included studies

Study ID	Study design Study period	Participants Sample size	Notes
**a) Financial interventions**			
***Health worker payment methods***			
Lo 2008 [22]	ITS Study period: 2001 to 2005	Country: Taiwan Pregnant women	Baseline (control group) CS rate: 29% Outcomes assessed: CS
Keeler 1996 [23]	ITS Study period: Data set used – 12 months before and 12 months after May 1993	Country: USA 11,767 deliveries (5255 cases for the 12 months before and 6515 cases for the 12 months afterwards)	Baseline (control group) CS rate: 25.3% Outcomes assessed: CS
Liu 2007 [24]	Interrupted time series analysis Study Period: Stage 1: May 1989; February 1996 Stage 2: 2001 to 2003	Country: Taiwan Participants: Stage 1: All women who gave birth between May 15 and 17, 1989 (1610) and February 12–16, 1996 (3546). Stage 2: All women who gave birth between 2001 and 2003	Baseline CS rate: 33 to 35% Outcomes assessed: overall CS
***Health organization payment methods***			
*Diagnosis-related group (DRG) payment systems*			
Kim 2016 [25]	Controlled before-after study Study period: 2011 to 2014	Country: South Korea Participants: 1,289,989 delivery cases in 674 hospitals	Baseline CS rate: 37% Outcomes assessed: CS
Lee 2007 [26]	Retrospective cohort study Study period: January – September 2003	Country: South Korea Participants: 179,222 patients (106,406 Diagnosis-Related Group (DRG) patients)	Baseline CS rate in Korea: 40.5% Outcomes assessed: overall CS
*Global budget payment (GBP) systems*			
Chen 2014 [27]	Retrospective pre-post reform case study Study period: May 2003 to April 2008.	Country: Taiwan Participants: 1,003,412 hospital admissions of women (18 to 45 years) for delivery, of which 1/3 were caesarean sections (5.6% of which were elective)	Baseline CS rate: 30.6% Outcomes assessed: overall CS, elective CS
Kozhimannil 2018 [28]	ITS Study period: 2006 to 2012	Country: USA Participants: 671,177 total maternal birth records (*N* = 25,080 in policy group, and *N* = 646,097 in control group)	Baseline CS rate: 22.8% Outcomes assessed: overall CS, childbirth hospitalization costs, maternal morbidity
Liu 2013 [29]	Interrupted time series analysis Study period: June 2001 to August 2010	Country: Taiwan Participants: 35,616 deliveries, including 12,831 CS. All pregnant women who delivered babies between June 2001 and August 2010 at Chang Gung Memorial Hospital in Linkou, Taiwan.	Baseline CS rate: 35.1% Outcomes assessed: overall CS, primary CS, repeat CS, VBAC
*Case-based payment system*			
Tsai 2006 [30]	Uncontrolled Before-after study Study period: “Vaginal Birth after Caesarean Section” (VBAC) case payment program implemented on April 1, 2003.	Country: Taiwan Physicians practicing VBAC. The data used in the study were derived from the health care system in Taiwan, including four of the system’s hospitals, 30 obstetric attendings, and 2246 gravidas with a previous caesarean section delivery under the attending physician’s care.	Baseline CS rate: unclear (full text article not available) Outcomes assessed: VBAC
*Cap-based payment system*			
Misra 2008 [31]	Design: Pre-post study using a comparison group with Maryland State inpatient databases. Pregnant women enrolled in Medicaid managed care were compared pre-implementation and post implementation with pregnant women delivering babies under private insurance. Study period: 1995 and 2000	Country: USA Participants: 128,743 births identified through Maryland State inpatient databases. 63,570 and 65,173 births in 1995 and 2000, respectively.	Baseline CS rate: 21.7% Outcomes assessed: primary CS, repeat CS, VBAC
Chen 2016 [32]	Retrospective pre/post reform case study Study period: January 2004 to December 2013. Reform instituted in 2009.	Country: China Participants: 6547 Caesarean delivery case records from a tertiary level hospital in Wuxi. 3240 cases were pre-reform and 3307 were post-reform.	The cap system does not reimburse hospitals for costs above the threshold (per capita) which disincentivizes doctors from prescribing unnecessary procedures. Baseline CS rate: 54% Outcomes assessed: rate of expenditure on CS compared to other patient services
*Other financial interventions*			
Karami 2018 [33]	ITS Study period: Intervention implemented in April 2014 (Monthly data C-section rate collected for a period of 53 Months – 25 months before and 28 months after the implementation of the HSEP from the information system of all 15 hospitals)	Country: Iran Participants: Fifteen hospitals affiliated to Ministry of Health and Medical Education (MoHME) in Kermanshah province.	Baseline CS rate: 43.4% Outcomes assessed: CS, hospitalization
**b) Regulatory and legislative interventions**			
Studnicki 1997 [34]	ITS Study period: Implemented fall 1992 Preintervention period: 1990–1992 Postintervention period: 1993	Country: USA Participants: Retrospective analysis of 366,246 total live births occurring in Florida hospitals during 1992 and 1993, before and after formal hospital certification of the implementation of the guidelines. Provider hospitals: were defined in the law as facilities in which 30 or more deliveries occurred annually that either were fully paid by state or federal funds administered by the state.	Baseline CS rate: 25.2% Outcomes assessed: primary CS, repeat CS
Yu 2017 [35]	Pre-post intervention study Study period: 2006 to 2014	Country: China Participants: 131,312 deliveries in 3 tertiary public hospitals between 2006 and 2014.	Baseline CS rate: 54.9% in China; 55.7% in the sample population Outcomes assessed: overall CS, caesarean delivery on maternal request (CDMR), Average annual growth rate (AAGR) of the overall CDMR.
**c) Other interventions**			
Snowden 2016 [36]	Retrospective cohort study Study period: 2008–2013	Country: USA Participants: 181,034 women who delivered in Oregon hospitals between 2008 and 2013, excluding 2011. 111,292 women delivered in the period before the hard-stop policy (2008–2010), and 69,742 women delivered after the rollout of the policy (2012–2013).	Baseline total CS rate: 26% Baseline early CS rate: 4% Outcomes assessed: CS, maternal morbidity, neonatal morbidity and mortality
Borem 2020 [37]	Interrupted time series (ITS) study Study period: 2014 to 2016 Baseline period: January to December 2014 to the year following the set-up period of *Projeto Parto Adequado* (PPA) or “Appropriate Birth” project; Full implementation period: January to December 2016	Country: Brazil Low risk women (nulliparous, term, singleton, vertex) in Brazilian hospitals. Twenty-eight hospitals enrolled in a 20-month quality improvement (QI) Collaborative that targeted low-risk pregnancies. 119,378 targeted deliveries in 13 intervention hospitals.	The primary aim of PPA was to increase vaginal delivery from a baseline of around 21.5 to 40% in the target population of 28 Brazilian hospitals over a 20-month intervention period, without worsening outcomes for mothers or infants. This flexible approach allowed adaptation to local priorities. Multiple strategies implemented simultaneously that are anchored in a learning system that constantly reassesses progress and makes modifications to the design. Baseline CS rate: range 21.5 to 40%. Outcomes assessed: vaginal deliveries, maternal and neonatal adverse events, NICU admissions

*CS* caesarean section, *NICU* neonatal intensive care admission, *CDMR* caesarean delivery on maternal request, *AAGR* average annual growth rate (AAGR), *VBAC* vaginal birth after caesarean section

### Interventions

Details of the interventions are summarised in Table 4.

**Table 4:** Description of included interventions

Study	Intervention	Details	
**a) Financial interventions**			
***Health worker payment methods***			
Lo 2008 [22]	Increase physician fees for vaginal birth after caesarean (VBAC) fee to the same level as caesarean section Increase in vaginal birth physician fees to that of caesarean section	National Health Insurance Taiwan equalised the fee for VBAC to that of a caesarean in April 2003. In May 2005, the fee for vaginal birth was raised to the equivalent of that of a caesarean section.	
Keeler 1996 [23]	Equalising physician fees for vaginal and caesarean delivery	Revision to fee schedule for obstetric and other procedures including equalising the fees for vaginal and caesarean sections.	
Liu 2007 [24]	National Health Insurance (NHI)	National Health Insurance (NHI) which equalized price for CS and vaginal delivery implemented in 1995.	
***Health organization payment methods***			
*Diagnosis-related group payment system*			
Kim 2016 [25]	Diagnosis-related Group (DRG) payment system	Diagnosis-related Group (DRG) payment system with fixed reimbursement for physicians regardless of cost of CS procedure. To promote vaginal delivery, the medical fee for vaginal birth increased by 50%, raising per diem profits above those of CS delivery and additional reimbursements were given for vaginal delivery of a patient over 35.	
Lee 2007 [26]	Diagnosis-Related Group (DRG) payment system.	Diagnosis-Related Group (DRG) prospective payment system (PPS) which sets a fixed fee for service. In a DRG group, the fee difference between CS and vaginal delivery is less than in a fee-for-service (control) system (in the DRG system the fee for CS was less than 2 times that for vaginal delivery. In the fee-for-service, the fee for CS was 2.7 times that for vaginal delivery). Three set DRG codes: CS, vaginal delivery with complication, vaginal delivery without complication. The fee for a set code is determined by the severity of complication/comorbidity index.	
*Global budget payment (GBP) system*			
Chen 2014 [27]	Global fee for obstetric services, increasing reimbursement for vaginal delivery to be equal to CS Co-payment when CS not indicated	Policy I: Financial incentives to encourage vaginal delivery through a global fee for obstetric services and increasing reimbursement for vaginal delivery to be equal to that of caesarean sections. Policy II: Copayment when caesarean section is not medically indicated (aimed to reduce the demand for elective caesarean procedure).	
Kozhimannil 2018 [28]	Global fee for uncomplicated deliveries (regardless of mode)	Single blended payment rate for uncomplicated births (regardless of mode of delivery). Before the policy, facility fees were $3144 and $5266 for uncomplicated vaginal and caesarean births, respectively. As of October 1, 2009, the policy changed the rate to $3528 for uncomplicated births, regardless of mode of delivery.	
Liu 2013 [29]	Global Budget System Hospital-based Self-Management (HBSM)	Global Budget System (GBS) in July 2002. This entails direct government funding for hospitals and by extension cost-reduction and elimination of unnecessary services. Hospital-based Self-Management (HBSM) in August 2005 involves post-operative peer reviews and audits to reduce medical service costs incurred by CS.	
*Case-based payment system*			
Tsai 2006 [30]	Vaginal birth after caesarean section (VBAC) case payment program	Vaginal birth after caesarean section (VBAC) case payment program introduced by Taiwan’s Bureau of National Health Insurance (BNHI).	
*Cap-based payment system*			
Misra 2008 [31]	Cap based payment system	The HealthChoice managed care program (mandatory for Medicaid recipients, and have risk-adjusted capitation rates designed to individualize care and reduce unnecessary CS rates.	
Chen 2016 [32]	Cap-based maternity insurance scheme (MIS)	Cap-based maternity insurance scheme (MIS) • Limits unnecessary expensive procedures by not reimbursing hospitals above price of cap. Patients no longer pay upfront. The cap system does not reimburse hospitals for costs above the threshold (per capita) which disincentivizes doctors from prescribing unnecessary procedures.	
***Other financial interventions***			
Karami 2018 [33]	Financial incentive and free vaginal delivery policy	Financial incentive and free vaginal delivery policy • Health Sector Evolution Plan (HSEP) reform provided free-of-charge inpatient services for vaginal delivery and offered financial incentives for providers to promote vaginal rather than CS delivery.	
**b) Regulatory and legislative interventions**			
Studnicki 1997 [34]	Legislatively imposed practice guidelines	Legislatively imposed practice guidelines • Mandated that practice guidelines regarding caesarean section deliveries be disseminated to obstetric physicians. • The law also required that peer review boards at hospitals be established to review caesarean deliveries and that the exact dates of implementation of the guidelines be reported to a state agency. The provider hospitals were required to provide copies of the guidelines to obstetric physicians and other persons appropriately credentialed to perform caesarean deliveries, establish a peer review board to review caesarean deliveries and ensure that its findings are shared, incorporate the peer review board reviews and reports into the hospital’s quality assurance monitoring and peer review process, and to report to the state Agency for Health Care Administration (AHCA) the dates of the implementation of the practice parameters and the initial meeting of the peer review board.	
Yu 2017 [35]	Multifaceted institutional and policy interventions.	**Institutional interventions** Health education (face-to-face weekly educational meetings between patients and hospital staff, training for obstetricians and midwives) painless delivery introduction, and doula care. **Policy interventions** 1. Development plans • The Regulation for the Management of Maternal Health Care and the Norms of Maternal Health Care Encourage mothers to choose vaginal delivery; Should strictly control indications for caesarean section (CS); Should strictly control caesarean delivery on maternal request (CDMR). • The Project of Maternal and Child Health During the 12th 5 Year Plan in Zhejiang Province Reduce the CS rate in Zhejiang Province. • The Development Plan for Women in Wenzhou Enhance health education about maternal health; Popularize knowledge about perinatal health care; Reduce the CS rate in Wenzhou area. 2. Evaluation criteria Medical Quality Management and Control Indicators for Tertiary Comprehensive Hospitals. The CS rate was included among patient safety indicators.	
c) **Other interventions**			
Snowden 2016 [36]	Oregon “hard-stop” policy limiting elective inductions and caesarean deliveries before 39 weeks of gestation	Oregon “hard-stop” policy limiting elective inductions and caesarean deliveries before 39 weeks of gestation. Introduced by the Oregon Perinatal Collaborative in 2011. The hard-stop policy limited early-term deliveries by requiring review and approval for any delivery without documented indication before 39 weeks of gestation (in contrast with other approaches, e.g., “soft-stop” policies, which give providers more discretion).	
Borem 2020 [37]	Quality improvement initiative: “Appropriate Birth”	Quality improvement initiative: *Projeto Parto Adequado* (PPA) or “Appropriate Birth.” comprised of four change packages: 1. Leadership **Coalition of major stakeholders aligned around primacy of safe mother, safe baby.**	
		Drivers of change	Change concept
		Alignment of financial incentives to hospitals and health plans. Drive change and remove barriers to create a learning and culture improvement. Engaged, activated community expecting best, safest care.	Leaders, champions, front line with the skills to do continuous improvement. Educate senior leaders, providers, community and patients about the benefits of physiologic birth. New contract between payers and providers creating incentives for quality and safety. New contract between health plan/hospital creating incentives for quality, safety and normal birth. Activate the community.
		2. Pregnant women **Empower pregnant women and their families to choose the care that is right for them (ensure readiness for normal birth).**	
		Drivers of change	Change concept
		Adequate information, based on evidence to support the best choice. Co-design and shared decision. Retake ownership of labor.	Educate and instruct families and pregnant women to new care model. Public campaigns. The intangible aspects of being a mother - delighting the pregnant women and families. Listening to mother and families.
		3. Healthcare system **New care model to accommodate the longer time frame of normal physiologic birth.**	
		Drivers of change	Change concept
		Perinatal redesigning. Confident and competent caregivers can support natural birth. Supportive environment for clinicians promotes “joy in work”. Shared care for mother-child unit. Reliable implementation of best clinical practice	Protocols and standardization for perinatal care. Physical space redesign (Ambiance for normal birth – Delivery Rooms). Invest resources to conquer healthy work environment. Well trained team to assist the deliveries. Multi-professional team assisting all pregnancy phases. Protocols and standardization for delivery and postpartum.
		4. Information **Data systems that support learning.**	
		Drivers of change	Change concept
		Transparency. Select measures to reflect quality and safety.	Create the capability to collect reliably information - measures and results. Feedback to professional teams, patients and families. Establish some quality and safety measures, report them to the providers and general public.

#### Financial interventions

Twelve studies published between 1996 and 2019 assessed financial interventions [22–33].

Study designs were varied: controlled before-after study [25], interrupted time series studies [22–24, 28, 29, 33]; uncontrolled before-after study [30]; pre-post comparative studies [27, 31, 32]; and retrospective cohort studies [26].

Ten studies were done in high-income countries: USA [23, 28, 31], Taiwan [22, 24, 27, 29, 30], South Korea [25, 26]. Two studies were done in middle-income countries: China [32] and Iran [33]. There were no studies from low-income countries.

The specific financial interventions were:

- *Health worker payment methods:* Equalising physician reimbursement fees for vaginal and caesarean delivery [22–24].
- *Health organization payment methods:* diagnosis-related group (DRG) payment system [25, 26]; global budget payment (GBP) system [27–29]; case-based payment system [30]; and cap-based payment system [31, 32].
- *Other financial interventions:* Financial incentive and free vaginal delivery policy [33].

#### Regulatory and legislative interventions

Two studies assessed regulatory and legislative interventions: interrupted time series study (USA) [34] and pre-post intervention study (China) [35].

The specific regulatory and legislative interventions were: legislatively imposed practice guidelines [34]; and multifaceted institutional and policy interventions [35].

#### Other interventions

Two studies, retrospective cohort study (USA) [36] and interrupted time series study (Brazil) [37], assessed the following interventions: “hard stop” policy limiting elective inductions and caesarean births [36]; and multifaceted quality improvement initiative (“Appropriate Birth”) [37].

### Effects of interventions

Details of the effects of the interventions and certainty of evidence ratings are summarised in Table 5.

**Table 5:** Effects of interventions

Study Intervention	Quality assessment					Effects of interventions	Effect on CS	Certainty (GRADE)
	Risk of bias	Inconsistency	Indirectness	Imprecision	Other aspects			
**a) Financial interventions**								
***Health worker payment methods***								
Lo 2008 [22] Increase physician fees for vaginal birth after caesarean (VBAC) fee to the same level as caesarean section Increase in vaginal birth physician fees to that of caesarean section	Serious^d^	Single study	Not serious	Not serious	None	The change in the level of total CS rates following the rise in VBAC fees was −1.68 (95% CI −2.3 to −1.07); the change in slope was −0.004 (95% CI −0.05 to 0.04)^e^ The change in the level of total CS rates (for all indications and order of birth) following the rise in vaginal birth fees was 1.19 (95% CI − 0.01 to 2.40) and the change in slope was − 0.43 (95% CI − 0.78 to − 0.09)^e^	CS decreased	⊕⊖⊖⊖ VERY LOW^d^
Keeler 1996 [23] Equalising physician fees for vaginal and caesarean delivery	Serious^d^	Single study	Not serious	Not serious	None	CS rates for non-breech deliveries decreased by 1.2% (22.5% before reform versus 21.3% after reform)	Effect on CS unclear	⊕⊖⊖⊖ VERY LOW^d^
Liu 2007 [24] National Health Insurance (NHI) which equalized price for CS and vaginal delivery	Not serious	Single study	Not serious	Not serious	None	The percentage of pregnant women who received cesarean sections increased by 6.3% after NHI, from 24.2 to 30.5%, but after controlling for other variables NHI was not found to have a significant impact on the rate.	No change in CS	⊕⊕⊖⊖ LOW
***Health organization payment methods***								
*Diagnosis-related group (DRG) payment systems*								
Kim 2016 [25] Diagnosis-related Group (DRG) payment system	Not serious	Single study	Not serious	Not serious	None	Longer DRG adoption period was associated with a lower odds of CS (OR 0.997, 95% CI: 0.996 to 0.998). Hospitals that underwent mandatory adoption of the DRG system performed 216,633 (37.3%) deliveries by CSs, whereas hospitals that underwent voluntary adoption of the DRG system performed 260,676 (36.8%) deliveries by CSs	CS decreased	⊕⊕⊖⊖ LOW
Lee 2007 [26] Diagnosis-Related Group (DRG) payment system	Not serious	Single study	Not serious	Not serious	None	No significant differences in CS rates between providers in DRG and fee-for-system (control) groups after controlling for organizational variables.	No change in CS	⊕⊕⊖⊖ LOW
*Global budget payment (GBP) system*								
Chen 2014 [27] Global fee for obstetric services, increasing reimbursement for VD to be equal to CS, co-payment when CS not indicated	Not serious	Single study	Not serious	Not serious	None	For women under 30 and over 45, the overall caesarean section rate increased following implementation of the global fee (18.8 to 19.4% for women aged 20, 27.6 to 28.3% for women aged 25, and 9.0 to 11.8% for women aged 45). The elective caesarean rate decreased for women under 30 (1.4 to 1.1% for 20-year-olds, and 2.1 to 1.7% for 25-year-olds) following the global fee intervention. Following the copayment intervention, elective caesarean sections increased for all age groups; mothers aged 20 had the highest odds (1.51) of electing for cesarean after the intervention (as compared to before the intervention). There was no statistically significant effect of either policy change on the elective caesarean rate for women over 40	Mixed-effects	⊕⊕⊖⊖ LOW
Kozhimannil 2018 [28] Global fee for uncomplicated deliveries (regardless of mode)	Not serious	Single study	Not serious	Not serious	None	Overall CS decreased 3.2 percentage points, with a 0.27 percentage points decrease per quarter (*p* = 0.01). The cost of childbirth hospitalizations decreased by $425.8 and continued to drop $95.0 per quarter (*p* < 0.001). No significant effects on maternal morbidity.	CS decreased	⊕⊕⊖⊖ LOW
Liu 2013 [29] Global Budget System (GBS) Hospital-based Self-Management (HBSM)	Not serious	Single study	Not serious	Not serious	None	No significant change in total CS rate. Primary CS rate increased from 23.6 to 26.9% post-GBS (from 2001 to 2002), but repeat CS decreased from 95.3 to 87.8%. VBAC increased from 4.8 to 12.2% in the same period. There were no significant changes after HBSM was introduced.	No change in CS	⊕⊕⊖⊖ LOW
*Case-based payment system*								
Tsai 2006 [30] Vaginal birth after cesarean section (VBAC) case payment program	Serious^b^	Single study	Not serious	Not serious	None	After implementation of VBAC case payments, the VBAC rates at the sampled hospitals increased 6.06% (p < 0.001).	VBAC increased	⊕⊖⊖⊖ VERY LOW^b^
*Cap-based payment systems*								
Misra 2008 [31] Cap-based payment system (HealthChoice managed care program with risk-adjusted capitation rates designed to individualize care and reduce unnecessary CS rates)	Not serious	Single study	Not serious	Not serious	None	Caesarean section increased as a proportion of all births (from 21.1 to 24.0%) and VBAC decreased from 4.7 to 3.6% during the same period.	CS increased VBAC decreased	⊕⊕⊖⊖ LOW
Chen 2016 [32] Cap-based maternity insurance scheme	Serious^d^	Single study	Not serious	Not serious	None	While all medical expenditures increased over time, the rate of expenditure on CS decreased as compared to other patient services in the hospital. The average annual growth rate for CS medical expenditures was significantly lower than that of inpatient and outpatient expenditures (3.8% vs. 8.3 and 13.0%).	Changes in CS not reported	⊕⊖⊖⊖ VERY LOW^d^
***Other financial interventions***								
Karami 2018 [33] Financial incentive and free vaginal delivery policy (financial incentives for providers to promote vaginal delivery rather than CS; free-of-charge inpatient services for VD)	Not serious	Single study	Not serious	Not serious	None	The proportion of caesarean sections decreased dramatically (−11.0%, *p* = 0.044) in the first month post-intervention, but overall the rate of caesarean sections increased by 0.0017% per month in the post-intervention period. Hospitalizations increased following the intervention (0.70 per 10,000, *p* < 0.01).	CS increased	⊕⊕⊖⊖ LOW
**b) Regulatory and legislative interventions**								
Studnicki 1997 [34] Legislatively imposed practice guidelines	Not serious	Single study	Not serious	Not serious	None	Caesarean sections decreased as a whole, but this trend was apparent prior to implementation of the guidelines. Repeat caesarean sections decreased by a greater magnitude (5.7%) following the intervention, suggesting that the guideline program may have a greater impact on repeat caesarean sections.	CS decreased	⊕⊕⊖⊖ LOW
Yu 2017 [35] Multifaceted institutional and policy interventions.^c^	Not serious	Single study	Not serious	Not serious	None	After institutional interventions were introduced, the overall CDMR rate increased from 15.8 to 16.3% (*p* = 0.053), but the average annual growth rate (AAGR) of the overall CDMR rate quickly declined from 20.1% to −4.3%. The overall CS rate declined by 1.29% from 2006 to 2008 and 2009–2010. The AAGR decreased from 0.29% to −6.73% over the same period. The overall CS rate decreased from 54.42 to 46.16% (*p* < 0.001). The AAGR of the CDMR rate decreased from − 4.30% to − 14.77% after policy interventions were applied. Post-intervention the CDMR, AAGR, the probability of performing CS, and the probability of a woman electing for CS decreased.	CS decreased	⊕⊕⊖⊖ LOW
**c) Other interventions**								
Snowden 2016 [36] “Hard-stop” policy limiting elective inductions and cesarean deliveries before 39 weeks of gestation	Not serious	Single study	Not serious	Not serious	None	The odds of overall elective caesarean section remained the same in the post-policy period compared with the pre-policy period (OR 1.00, 95% CI, 0.97 to 1.03). The odds of chorioamnionitis (OR 1.94, 95% CI 1.80 to 2.09) and blood transfusion (OR 1.42, 95% CI 1.20 to 1.67 were elevated in the post-policy period compared with the pre-policy period. The odds of stillbirth (OR 1.20, 95% CI 0.88 to 1.63) and neonatal death (OR 1.34, 95% CI 0.87 to 2.07) remained the same in the post-policy period compared with the pre-policy period.	No change in CS	⊕⊕⊖⊖ LOW
Borem 2020 [37] Quality Improvement Initiative (“Appropriate Birth”)	Not serious	Single study	Not serious	Not serious	None	Quality improvement initiative was associated with a 62% increase in the rate of vaginal deliveries (vaginal deliveries increased from 21.5% (95% CI 15.8 to 29.2%) in 2014 to 34.8% (95% CI 28.9 to 41.9%) in 2016, a relative increase of 1.62 (95% CI 1.27 to 2.07, p < 0.001), equivalent to a 62% increase in VD). Rates of adverse events^a^ (IRR 1.13, 95% CI 0.88 to 1.46) and NICU admissions (IRR 1.13, 95% CI 0.91 to 1.4).	Vaginal births decreased	⊕⊕⊖⊖ LOW

*Abbreviations*: *ITS* interrupted time series study, *OR* odds ratio, *CI* confidence interval, *VBAC* vaginal birth after cesarean section, *CS* caesarean section, *VD* vaginal deliveries, *NICU* neonatal intensive care unit, *IRR* incidence rate ratio

^a^Maternal death, intrapartum or neonatal death > 2.5 kg, uterine rupture, maternal admission to intensive care unit, birth trauma (neonatal), return to operating room, admission to NICU > 2.5 kg for > 24 h, Apgar score < 7 at 5 min, blood transfusion, 3rd or 4th degree perineal tear

^b^Downgraded one level for serious risk of bias due to possible confounding (lack of concurrent control group)

^c^Institutional interventions (face-to-face weekly educational meetings between patients and hospital staff, training for obstetricians and midwives, introduction of painless delivery, doula care) and policy interventions (policies to decrease high CS rate by controlling caesarean delivery on maternal request (CDMR) rate, requirement for health providers to encourage vaginal delivery and rigorously control indications for CS, inclusion of CS rate among patient safety indicators)

^d^Downgraded one level for serious risk of bias (due to possible confounding of outcome, unclear whether the intervention occurred independently of other changes over time)

^e^Two standardised effect sizes are obtained from ITS analysis: a change in level (also called ‘step change’) and a change in trend (also called ‘change in slope’) before and after the intervention. Change in level = difference between the observed level at the first intervention time point and that predicted by the pre-intervention time trend; change in trend = difference between post- and pre-intervention slopes. A negative change in level and slope indicates a reduction in CS rate

#### Financial interventions

##### Equalising physician reimbursement fees for vaginal and caesarean delivery

The three studies that examined programs involving equalising physician fees for vaginal and caesarean deliveries reported mixed-effects on CS rates [22–24]. Two studies found little or no difference in CS rates following equalising fees by National Health Insurance (NHI) in Taiwan [22, 24]. One of the studies [24] reported a non-significant change in CS from 24.2 to 30.5%. In the other study [22], the change in the level of total CS rate was − 1.68 (95% CI − 2.3 to − 1.07) while the change in trend was − 0.004 (95% CI − 0.05 to 0.04). Effects of equalising physician fees was unclear in the third study conducted in USA [23]). In this study CS rates decreased by 1.2% but absolute numbers for the change were not reported (re-analysis to determine statistical significance was therefore not possible). The certainty of evidence varied from very low [22, 23] to low [24].

##### Diagnosis-related group payment system

The two studies from South Korea that assessed diagnosis-related group (DRG) payment systems found mixed-effects on CS rates [25, 26]. The first study [25] explored the impact of a DRG payment system with fixed reimbursement for physicians regardless of the cost of the caesarean procedure. The findings showed that, in hospitals that voluntarily adopted this practice, longer periods of DRG adoption were associated with lower odds for CS (OR 0.997, 95% CI 0.996 to 0.998). However, hospitals that underwent mandatory DRG adoption had higher CS rates (37.3% vs. 36.8%). The second study [23, 26] found no differences in CS rates between DRG payment systems operated by voluntarily participating hospitals compared to fee-for-service systems. The certainty of evidence was low in both studies.

##### Global budget payment system

Mixed-effects were observed across groups of women of different ages following implementation of a global fee policy for caesarean and vaginal deliveries and co-payment from patients for non-medically indicated caesareans in Taiwan (effect estimates presented in Table 5) [27]. In USA [28], a global payment policy (i.e. a single facility or professional services payment regardless of delivery mode) resulted in a decrease in overall CS by 3.2 percentage points. An additional study from Taiwan [29] found no significant effect on CS following implementation of a GBS and Hospital-Based Self-Management (HBSM) policy which involved post-operative peer reviews and audits of CS (effect estimates presented in Table 5). The certainty of evidence in each of the studies was low.

##### Case-based payment system

Vaginal birth after caesarean section (VBAC) increased by 6.1% (*p* < 0.001) after implementation of a case-based payment system in one study conducted in Taiwan [30]. The certainty of evidence was very low.

##### Cap-based payment system

Implementation of a cap-based payment system (i.e. HealthChoice managed care program) resulted in an increase in CS (from 21.1 to 24.0%) and a decrease in VBAC (from 4.7 to 3.6%) in one study from USA [31]. An additional study (China) [32] assessed the effect of a cap-based Maternity Insurance Scheme (MIS) designed to limit unnecessary procedures by refusing to reimburse hospitals above the price of a cap. While all medical expenditures increased over time, the rate of expenditure on CS decreased as compared to other patient services in the hospital. Changes in CS rates were not reported. The certainty of evidence was low in both studies.

##### Financial incentive and free vaginal delivery policy

One study conducted in Iran [33] reported an increase in CS following implementation of a financial incentive for providers to promote vaginal delivery combined with free vaginal delivery policy (rate of CS increased by 0.0017% per month in the post-intervention period). The certainty of evidence was low.

#### Regulatory and legislative interventions

Studied regulatory and legislative interventions (comprising legislatively imposed practice guidelines for physicians [34] and multi-faceted strategy which included policies to control caesarean delivery on maternal request (CDMR) [35]) were found to reduce CS rates. In one of the studies (USA) [34], repeat CS decreased by a greater magnitude (5.7%) following implementation of legislatively imposed practice guidelines on CS for obstetricians (which included the establishment of peer review boards at hospitals for quality control). In the other study (China) [35] the overall CDMR rate decreased from 54.4 to 46.2% (*p* < 0.001) following implementation of the policy interventions. The certainty of evidence was low in both studies.

#### Other interventions

One study (USA) examined the effect of a “Hard stop” policy limiting elective inductions and caesarean births before 39 weeks of gestation [36]. In order to limit early-term deliveries, the policy required a review and approval for any delivery without documented indication prior to 39 weeks of gestation. The study found no effect on elective CS (OR 1.00, 95% CI 0.97 to 1.03). An additional study (Brazil) [37] assessed the effect of a multifaceted quality improvement initiative (“Appropriate Birth”), that among other change packages, included new contracts between payers and providers and between health plan and hospital creating incentives for quality and safety. Vaginal births increased following implementation of the quality improvement initiative (relative increase of 1.62, 95% CI 1.27 to 2.07). The GRADE certainty of evidence was low in both studies.

## Discussion

### Summary of main results

This scoping review identified diverse financial and legislative strategies intended to reduce CS rates, mostly from high income countries. Most of the studies assessed reimbursement strategies for health providers and organisations. Only two studies assessed regulatory and legislative strategies. Effects of the interventions were inconsistent across settings (for example, a global payment policy comprising a single facility or professional services payment regardless of delivery mode resulted in a decrease in CS in USA [28], but no effect was found in Taiwan [29]). For some interventions, effects were contradictory given hypothesised mechanisms of action (e.g. increased CS rates following cap-based payment system in USA [31]). Overall, our confidence in these findings is low given limitations in the available evidence. It is plausible that confounding due to observational designs impacted on the observed effects (for example, it was unclear in most of the studies whether the intervention occurred independent of other changes over time).

### Limitations of the evidence

None of the studies was done in a low-income country. Limited data was available on maternal and neonatal outcomes and health care resource utilisation, including impact on medical costs. This represents a significant gap in knowledge about the studied interventions.

The summarised evidence is mostly drawn from single studies assessing distinct interventions with diverse study designs. We were therefore unable to pool outcome data in order to increase sample size. Also, most of the evaluations were conducted a few years after the introduction of the interventions or policy reforms. Longer-term evaluations would be beneficial to reliably determine the impact and sustainability of the interventions.

Most of the studies do not provide sufficient details about the different components of the interventions to make it possible to determine their mechanisms of action, and to facilitate replication in other contexts. Evaluating and reporting effects of different intervention components can inform the extent to which various components influence CS rates and, therefore, inform optimal design of financial and legislative interventions.

The reliability of the effects of the interventions on CS rates depend on the quality of data available in the study sites. Coding errors limit reliability and validity of measurements [31]. In addition, coding practices in diagnosis-related payment systems may vary limiting comparisons across hospitals and sites. Thus, robust, standardized health information system for routine data collection are needed to support reliable monitoring of the impact of studied financial and regulatory interventions.

It was not possible to determine the appropriateness or indications of the reported CS, as none of the studies reported clinical data on the CS performed. It is therefore unclear whether the reported changes in CS rates were all in those considered unnecessary. Given these limitations, the review findings should be interpreted cautiously. Lastly, although our searches were relatively comprehensive, it is possible that we did not identify some relevant studies, particularly as no grey literature sources were searched.

### Implications for future research

The findings of this review have important implications for future research. First, the impact of different financial, regulatory and legislative strategies should be explored using larger sample sizes, for example, by analyses of pooled health facility survey or Multiple Indicator Cluster (MICS) data from different countries. Second, there were inconsistencies in the way that studies measured and reported CS, making it difficult to compare findings of the studies. More systematic and consistent reporting of CS would be useful to aid synthesis and interpretation of findings across studies and countries. For this, WHO proposes Robson classification system as a global standard [38]. Third, for financial reforms that involve a higher reimbursement fee for vaginal compared to caesarean deliveries, a monitoring system for vaginal deliveries is needed, given possible unintended consequences of inappropriate vaginal deliveries among high risk groups for CS. There is a continual need for replication of studies in different countries and populations with different health needs, particularly as most of the interventions were evaluated in single studies with weak study designs.

None of the 16 included studies utilised a randomised design. This suggests that there might be challenges to using randomised study designs for evaluating financial, legislative and regulatory interventions (e.g. randomisation of policy reforms may not be feasible in practice). Changes in payment systems or legislative policies are often implemented suddenly or nationwide, which prevents the use of randomised studies. In addition, identification of comparable control groups remains a challenge given inherent differences in contexts such as health systems, policy environments and population groups. Given these methodological challenges, balancing pragmatism and research rigour is encouraged in evaluating the effects of financial, regulatory and legislative interventions [39]. Similarly, new methods for assessing the quality of evidence optimised for studied and related policy interventions should be developed (existing systems such as GRADE may not be reliable given the highlighted methodological challenges in the conduct of primary studies). Well conducted controlled before and after designs and interrupted time series, with appropriate analysis to control for contextual factors and possible confounders, appear feasible alternatives to randomised designs.

### Key lessons learned

A number of lessons relevant for the design and implementation of financial and legislative interventions emerged from the included studies. Several factors may have influenced the reported effects of the interventions: for example, it is possible that the 21% shift in payments in fee equalisation insurance reform in USA [23] may not have been enough to change physician behaviour to reduce CS. Given this, it is important that appropriate reimbursement is provided to healthcare providers to reduce economic incentives on decisions regarding the delivery method. If providers are reimbursed according to their performance, clinical condition would be considered to be more important when deciding on the mode of delivery, and this could play a role in decreasing the high rate of CS [25]. Also, mere dissemination of guidelines by a state agency did not achieve the magnitude and specificity desired without an explicit implementation program, in the legislatively imposed practice guidelines in USA [34]. Thus, dissemination of guidelines should be accompanied by systematic and detailed evidence-based implementation plan to improve effectiveness. These findings emphasize the need for multifaceted interventions targeting all key stakeholders to address caesarean over-use.

## Conclusions

The available evidence on the effects of financial and regulatory strategies intended to reduce unnecessary CS is inconclusive given inconsistency in effects and low quality of the available evidence. More rigorous studies and new ways of assessing the impact of financial and legislative interventions intended to reduce unnecessary CS are needed.

## Supplementary information

**Additional file 1.** Search strategy.

## Data Availability

All data generated or analysed during this study are included in this published article and its additional files.

## Abbreviations

GBP: Global budget payment
CBA: Controlled before-after study
CDMR: Caesarean delivery on maternal request
CINAHL: Cumulative index to nursing and allied health
CS: Caesarean section
DRG: Diagnosis-related group
GBP: Global budget payment
GIM: Global Index Medicus
GRADE: Grading of recommendations, assessment, development and evaluation
HBSM: Hospital-based self-management
ITS: Interrupted time series study
LILACS: Latin America and the Caribbean Literature on Health Sciences
MICS: Multiple indicator cluster
MIS: Maternity insurance scheme
NHI: National Health Insurance
PTSD: Post-traumatic stress disorder
RCT: Randomised controlled trial
VBAC: Vaginal birth after caesarean section
WHO: World Health Organization
