# Supplementary material for: Reducing unnecessary caesarean sections: scoping review of financial and regulatory interventions
Source: Reprod Health. 2020 Aug 31;17:133. doi: 10.1186/s12978-020-00983-y (PMC7457477; doi:10.1186/s12978-020-00983-y)
Supplement: Supplementary file 1 — Additional file 1. Search strategy. [file 12978_2020_983_MOESM1_ESM.docx]

Additional file 1. Search strategy

| MEDLINE (1946 to 6 June 2019 | | |
| --- | --- | --- |
| Concept | Search string | Results |
| #1 | "Natural Childbirth"[Mesh] OR "Vaginal Birth after Cesarean"[Mesh] OR "Cesarean Section"[Mesh] OR "normal childbirth" OR "normal birth" OR "vaginal birth" OR "vaginal childbirth" OR “Abdominal Deliveries”[TW] OR “C Section” [TW] OR “C Sections” [TW] OR Postcesarean[TW] OR Postcaesarean[TW] OR Cesarean[TW] OR Caesarean [TW] | 74677 |
| #2 | "Legislation" [Publication Type] OR "Legislation as Topic"[Mesh] OR "Legislation, Medical"[Mesh] OR  "Taxes”[MeSH] OR  "Social Control, Formal"[Mesh] OR  "Legal Case" [Publication Type] OR  "legislation  and  jurisprudence" [Subheading] OR “Fiscal Policy”[MeSH] OR “economics”[subheading] OR laws [TW]  OR law[TW] OR legislation[TW]  OR jurisprudence[TW]  OR legal [TW] OR tax[tiab] OR taxes[tiab] OR taxation[tiab] OR taxed[tiab] OR taxing[tiab] OR subsidy[tiab] OR subsidies[tiab] OR subsidized[tiab] OR subsidised [TIAB] OR incentive[tiab] OR incentives[tiab] OR discount[tiab] OR discounts[tiab] OR discounted[tiab] OR price[tiab] OR prices[tiab] OR priced[tiab] OR  fiscal [tiab] OR rebate[tiab] OR vouchers[tiab] OR rebates[tiab] OR levy[tiab] OR financial [TIAB] | 1231959 |
| #3 | #1 AND #2 | 3112 |
| #4-Human Filter | NOT ("animals"[MeSH] NOT "humans"[MeSH]) AND "humans"[MeSH] | 2914 |

| EMBASE (<http://www.embase.com>) 1974 to 6 June 2019 | | |
| --- | --- | --- |
| Concept | Search string | Results |
| #1 | 'Natural Childbirth'/exp OR 'vaginal delivery'/exp  OR 'Cesarean Section'/exp OR 'Vaginal Birth after Cesarean'/exp  OR "Abdominal Deliveries":ti,ab,de OR "C section":ti,ab,de OR "C sections":ti,ab,de OR Postcesarean:ti,ab,de OR Postcaesarean:ti,ab,de OR Cesarean:ti,ab,de OR Caesarean:ti,ab,de OR "natural childbirth" OR "normal childbirth" OR "normal birth" OR "vaginal birth" OR "vaginal childbirth" OR "vaginal delivery" | [131,012](https://www-embase-com.ezproxy-b.deakin.edu.au/) |
| #2 | 'Law'/exp OR 'medicolegal aspect'/exp OR 'Tax'/exp OR 'social control'/exp OR 'Fiscal Policy'/exp OR laws:ti,ab,de OR law:ti,ab,de OR legislation:ti,ab,de OR jurisprudence:ti,ab,de OR legal:ti,ab,de OR tax:ti,ab OR taxes:ti,ab OR taxation:ti,ab OR taxed:ti,ab OR taxing:ti,ab OR subsidy:ti,ab OR subsidies:ti,ab OR subsidized:ti,ab OR subsidised:ti,ab OR incentive:ti,ab OR incentives:ti,ab OR discount:ti,ab OR discounts:ti,ab OR discounted:ti,ab OR price:ti,ab OR prices:ti,ab OR priced:ti,ab OR fiscal:ti,ab OR rebate:ti,ab OR vouchers:ti,ab OR rebates:ti,ab OR levy:ti,ab OR financial:ti,ab | [973,901](https://www-embase-com.ezproxy-b.deakin.edu.au/?org.apache.catalina.filters.CSRF_NONCE=BB4CFD0D0F10BCDA5D401F30273C42B9) |
| #3 | #1 AND #2 | 4,172 |

| CINAHL (data inception to 6 June 2019) | | |
| --- | --- | --- |
| Concept | Search string | Results |
| #1 | MH "Childbirth+" OR MH "Vaginal Birth After Cesarean" OR MH "Cesarean Section+" OR MH "Vaginal Birth+" OR TI  Cesarean OR TI  Caesarean OR  TI (C-sections) OR  TI(C-section) OR TI (Abdominal Deliveries) OR TI (Abdominal Delivery) OR TI Postcesarean OR TI Postcaesarean OR TI (Post cesarean)  OR TI (Post caesarean) OR AB  Cesarean OR AB  Caesarean OR  AB (C-sections) OR  AB (C-section) OR AB (Abdominal Deliveries) OR AB (Abdominal Delivery) OR AB Postcesarean OR AB Postcaesarean OR AB (Post cesarean)  OR AB (Post caesarean) | 23,258 |
| #2 | MH "Legislation+" OR MH "Legislation, Medical+" OR MH "Taxes+" OR MH "Social Control+" OR laws OR law OR legislation OR jurisprudence OR legal OR TI tax OR AB tax OR TI taxes OR AB taxes OR TI taxation OR AB taxation OR TI taxed OR AB taxed OR TI taxing OR AB taxing OR TI subsidy OR AB subsidy OR TI subsidies OR AB subsidies OR TI subsidized OR AB subsidized OR TI subsidised OR AB subsidised OR TI incentive OR AB incentive OR TI incentives OR AB incentives OR TI discount OR AB discount OR TI discounts OR AB discounts OR TI discounted OR AB discounted OR TI price OR AB price OR TI prices OR AB prices OR TI priced OR AB priced OR TI fiscal OR AB fiscal OR TI rebate OR AB rebate OR TI vouchers OR AB vouchers OR TI rebates OR AB rebates OR TI levy OR AB levy OR TI financial OR AB financial | 499,472 |
| #3 | #1 AND #2 | 1,300 |

| GIM (Global Health Library), <http://www.globalhealthlibrary.net>  Database inception to 6 June 2019 | | |
| --- | --- | --- |
| Concept | Search string | Results |
| #1 | ((Cesarean ) OR (Caesarean ) OR (Cesareans ) OR (Caesareans ) OR  (C-sections) OR (C-section) OR (Abdominal Deliveries) OR (Abdominal Delivery) OR (Postcesarean)  OR (Postcaesarean)) | **11.486** |
| #2 | ((laws) OR (law) OR (legislation) OR (jurisprudence) OR (legal) OR (tax) OR (taxes) OR (taxation) OR (taxed) OR (taxing) OR (subsidy) OR (subsidies) OR (subsidized) OR (subsidised) OR (incentive) OR (incentives) OR (discount) OR (discounts) OR (discounted) OR (price) OR (prices) OR (priced) OR (fiscal) OR (rebate) OR (vouchers) OR (rebates) OR (levy) OR (financial)) | **59.252** |
| #3 - #1 AND #2 | ((Cesarean ) OR (Caesarean ) OR (Cesareans ) OR (Caesareans ) OR  (C-sections) OR (C-section) OR (Abdominal Deliveries) OR (Abdominal Delivery) OR (Postcesarean)  OR (Postcaesarean))  AND ((laws) OR (law) OR (legislation) OR (jurisprudence) OR (legal) OR (tax) OR (taxes) OR (taxation) OR (taxed) OR (taxing) OR (subsidy) OR (subsidies) OR (subsidized) OR (subsidised) OR (incentive) OR (incentives) OR (discount) OR (discounts) OR (discounted) OR (price) OR (prices) OR (priced) OR (fiscal) OR (rebate) OR (vouchers) OR (rebates) OR (levy) OR (financial)) | **211** |

| Ebsco MultiDisciplinary Databases (<http://www.ebsco.com>)  Database inception to 6 June 2019 | | |
| --- | --- | --- |
| Concept | Search string | Results |
| #1 | (SU "Vaginal Birth After Cesarean" OR SU "Cesarean Section" OR SU "Vaginal Birth" OR TI  Cesarean OR TI  Caesarean OR  TI “C-sections” OR  TI “C-section” OR TI “Abdominal Deliveries” OR TI “Abdominal Delivery” OR TI Postcesarean OR TI Postcaesarean OR TI “Post cesarean”  OR TI “Post caesarean”) AND (SU "Legislation" OR SU "Legislation, Medical" OR SU "Taxes" OR SU "Social Control" OR laws OR law OR legislation OR jurisprudence OR legal OR TI tax OR AB tax OR TI taxes OR AB taxes OR TI taxation OR AB taxation OR TI taxed OR AB taxed OR TI taxing OR AB taxing OR TI subsidy OR AB subsidy OR TI subsidies OR AB subsidies OR TI subsidized OR AB subsidized OR TI subsidised OR AB subsidised OR TI incentive OR AB incentive OR TI incentives OR AB incentives OR TI discount OR AB discount OR TI discounts OR AB discounts OR TI discounted OR AB discounted OR TI price OR AB price OR TI prices OR AB prices OR TI priced OR AB priced OR TI fiscal OR AB fiscal OR TI rebate OR AB rebate OR TI vouchers OR AB vouchers OR TI rebates OR AB rebates OR TI levy OR AB levy OR TI financial OR AB financial) | **1294** |
